# Brian M. Davies, MD, DPM, FRCP, FRCPsych, FRANZCP

**DOI:** 10.1192/bjb.2021.24

**Published:** 2021-12

**Authors:** Roger Glass

Formerly Cato Professor of Psychiatry, University of Melbourne, Victoria, Australia

**Figure:**
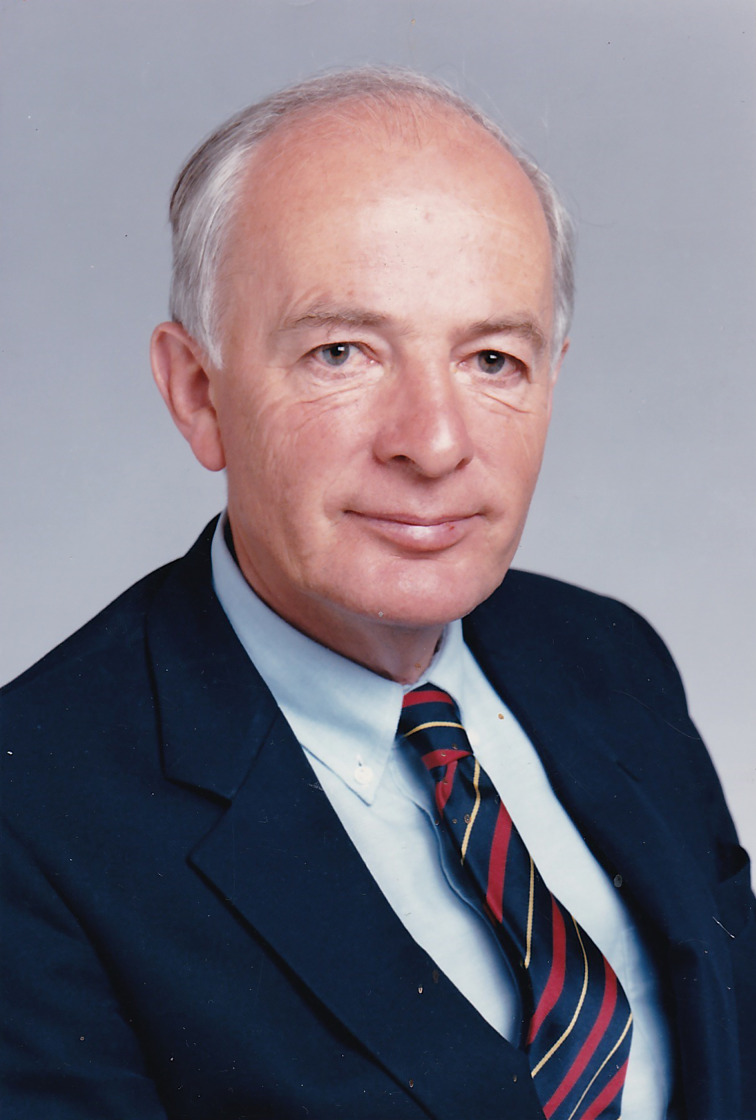

Professor Brian Davies, Victoria's first Professor of Psychiatry, died on 15 November 2020 at the age of 92. As a pioneer of academic psychiatry, he was regarded as a trail-blazer for Australian academic psychiatry who facilitated the training and career development of many successful colleagues. He was appointed to the foundation chair of the University of Melbourne's first academic Department of Psychiatry, taking up the Cato Chair in 1964. He rapidly established the department in the forefront of the specialty. The department that he led quickly gained an enviable reputation for wide-ranging quality research, excellent teaching, and state of the art treatment and caring for patients. To the end of his tenure 26 years later, he steadfastly focused his energies in three main areas: teaching students and postgraduates, service provision and facilitating quality research.

In his typically modest way, Brian attributed the fact that he was able to attract excellent people to accept positions in his department to ‘good fortune’ rather than the fast-developing reputation of his department as a centre of excellence. The Cato Chair became, arguably, the most prestigious Chair of Psychiatry in Australia, with staff receiving many Australian and New Zealand Commonwealth honours. Many leading psychiatrists, including George Szmukler, Edmond Chiu, David Ames, Graham Mellsop, Sidney Bloch and Graham Burrows, either received their early training or worked as psychiatrists and researchers in his department. His quiet, diffident and, at times, enigmatic nature resulted in a leadership style that enabled department staff to feel free to pursue their research passions while also being gently guided and supported yet nudged when necessary. He led not by acting as a loud front man, forever talking up the achievements of his ‘empire’, but more as an avuncular encourager and facilitator, open to good ideas but willing to guide and advise when required. The postgraduate psychiatry trainees in Brian's unit learned much from observing him assess patients, quickly and efficiently focusing in on the most salient aspects of their presentation and demonstrating his renowned clinical acumen. To many he became a mentor as well as a teacher. He expected much but also returned loyalty and support.

Brian played a leading role in the group of psychiatrists and business entrepreneurs who conceived the idea of building a modern private psychiatric hospital in Melbourne in 1975. He characteristically played down his pivotal role in setting up and conducting meetings that culminated in the opening of The Melbourne Clinic in 1978. The clinic became Australia's largest private psychiatric hospital. After retiring from the Cato Chair of Psychiatry in 1990, Brian continued to work in private practice, both at The Melbourne Clinic and elsewhere, finally retiring in 2009, aged 81.

Born on 8 June 1928 in Llanelli, South Wales, the son of a miner, Tom Davies, and Lilian (née Drake), a primary school teacher who was passionate about English literature, Brian followed his 6-years-older brother to Cardiff University Medical School. He graduated in 1950, his medical training having included just one lecture in psychiatry. He said: ‘I wasn't interested – in those days psychiatry was the last chapter in the medical textbooks – I just wanted to be a physician like my brother’. Having completed training as a physician, Brian asked senior colleagues for advice regarding what should be his next career step. ‘Do psychiatry training – psychiatry will be big in 4 years’ was the advice. He was able to secure a training position at the Maudsley Hospital in London, where he worked in the unit of Professor Sir Aubrey Lewis, an Australian psychiatrist who had become a towering figure in UK psychiatry. From 1956 to 1964 at the Maudsley and Bethlem Royal Hospital, Brian immersed himself in the newly emerging and exciting field of research into antidepressants. He published world-leading research on tricyclic and monoamine oxidase inhibitor antidepressants.^1^

Looking beyond the possibilities of a psychiatric career in Britain, he decided to apply instead for a more interesting academic post, just then advertised – that of Cato Professor of Psychiatry at the University of Melbourne. He, his wife and two children arrived in Melbourne in April 1964, after a 4-week sea journey. His outstanding ability was rapidly recognised and in 1968 he delivered the Beattie-Smith lecture, the highest honour that Melbourne psychiatry bestows on its leaders. His topic was: ‘Recent Studies of Severe Mental Depressive Illnesses’. He authored a classic student textbook, *An Introduction to Clinical Psychiatry*, which went through four editions from 1966 and remains well-known to a generation of Australian medical students and psychiatrists.

Brian had a quiet manner. He had very little ‘small talk’ and colleagues could sometimes feel that he kept them at a distance. This was more to do with his shy nature than any personal dislike. Persistence in pursuing issues with Brian was often rewarded with knowledgeable and helpful suggestions, advice and encouragement. In recent years he had delighted in reconnecting, at regular lunches, with many former work colleagues from his time as Cato Professor. He remained modest, continuing to be somewhat uncomfortable when publicly praised and never seeking special recognition or honours for himself.

Reading was a lifelong passion for Brian, his taste extending over many genres. He had a love for, and a deep knowledge of, Shakespeare. He also developed an enduring love of golf. He became a skilled and well-known player at Royal Melbourne Golf Club, actively participating until his last year.

In 1951, Brian married Rona (née Waters), a nurse, the start of over 67 years of loving marriage, sadly ended in 2019 with Rona's death. They had a daughter, Debra, and son, Gareth, together with three admiring grandchildren.

## References

[1] Rees L, Davies B. A Controlled Trial of Phenelzine (‘Nardil’) in the Treatment of Severe Depressive Illness. J Ment Sci 1961; 107: 560–6. 13740299 10.1192/bjp.107.448.560

